# Effects of predation pressure and resource use on morphological divergence in omnivorous prey fish

**DOI:** 10.1186/1471-2148-13-132

**Published:** 2013-06-27

**Authors:** Kristin Scharnweber, Kozo Watanabe, Jari Syväranta, Thomas Wanke, Michael T Monaghan, Thomas Mehner

**Affiliations:** 1 Department of Biology and Ecology of Fishes, Leibniz-Institute of Freshwater Ecology and Inland Fisheries, Berlin, Germany; 2 Department of Ecosystem Research, Leibniz-Institute of Freshwater Ecology and Inland Fisheries, Berlin, Germany; 3 Department of Civil and Environmental Engineering, Ehime University, Matsuyama, Japan; 4 Department of Biological and Environmental Sciences, University of Jyväskylä, Jyväskylä, Finland; 5 Department of Lake and River Fishery, Institute of Inland Fisheries, Potsdam, Germany

**Keywords:** Geometric morphometrics, AFLP, Stable isotope analysis, Gut content analysis, Shallow lakes, Predation, Outlier loci, Adaptive divergence, *Rutilus rutilus*, Predator induced morphological defense

## Abstract

**Background:**

Body shape is one of the most variable traits of organisms and responds to a broad array of local selective forces. In freshwater fish, divergent body shapes within single species have been repeatedly observed along the littoral-pelagic axes of lakes, where the structural complexity of near shore habitats provides a more diverse set of resources compared to the open-water zones. It remains poorly understood whether similar resource-driven polymorphism occurs among lakes that vary in structural complexity and predation pressure, and whether this variation is heritable. Here, we analyzed body shape in four populations of omnivorous roach (*Rutilus rutilus*) inhabiting shallow lakes. We tested the relationship between body shape, gradients of resources, predation pressure, and, in a subset of two lakes, diet composition. We used genome scans of 331 polymorphic AFLP markers to test whether there was a heritable component to the observed morphological diversification.

**Results:**

Body shape differed among lakes and was significantly correlated to differences in predation pressure. Roach from the lake with highest predation pressure were most divergent from the average body shape of all populations, characterized by a more streamlined body and caudally inserted dorsal fins; features that facilitate predator escape. Surprisingly, diet composition was not associated with morphology. AFLP analysis revealed weak genetic differentiation among lakes and no isolation by distance (IBD). Outlier analysis detected three loci under positive selection with differing frequencies in the four populations. General linear models did not support an association of lake-specific genotypes with morphological variation.

**Conclusion:**

Body shape was divergent among lakes, suggesting that processes previously reported from within single lakes may also be operating at the scale of whole lakes. We found no evidence for body shape being heritable, although sample size was small in these natural populations. Rather than habitat structure and diet, we conclude that predation had a stronger effect on the prevalence of local morphotypes. A variable morphotype facilitating the efficient uptake of a variety of spatially and temporarily scattered resources seems to be favored in these small aquatic systems.

## Background

Populations can exhibit divergent body morphologies in response to environmental cues and these differences may have a significant influence on the performance of individuals [1]. A common form of intraspecific divergence in body morphology is resource polymorphism, where morphological variation is associated with segregation in habitat and diet [2,3]. In freshwater fish, divergence of morphs has been frequently shown along the pelagic-littoral habitat axis of lakes (i.e., from the near shore to open-water habitats) [3]. Foraging in the open water is associated with a high search rate for widely distributed and conspicuous planktonic prey. A more streamlined body occurs in these morphs to facilitate a high attack speed. In contrast, foraging in the structurally complex littoral zones results in a lower search rate for more cryptic benthic prey. Thus, a deeper body supports higher manoeuvrability [4,5]. Fish species with coexisting pelagic and benthic morphs include three-spined stickleback (*Gasterosteus aculeatus*) [6-8], Eurasian perch (*Perca fluviatilis*) [9-11] and Arctic charr (*Salvelinus alpinus*) [12-14].

Predation pressure is another potentially selective force affecting the morphology of organisms. “Inducible defenses” [15] have been intensively studied in cladocerans [16,17] and mussels [18] and are known from a range of fish species. In the presence of predators, Crucian carp (*Carassius carassius*) develop a deeper body that increases the handling time by predators [19]. Pumpkinseed sunfish (*Lepomis gibbosus*) increase body depth and dorsal spine length when stimulated by permanent predation cues of walleye (*Sander vitreus*) [20]. Similar predator-induced responses have been described for perch [21].

Phenotypic variation can result from phenotypic plasticity [22,23] or from heritable adaptations to local selective forces [24-27]. To distinguish between phenotypically plastic and inherited variation, population genomics are widely used to test for the genetic basis of adaptive divergence [28]. Genome scans are an appropriate method to compare natural populations of non-model organisms that are nonetheless ecologically important, allowing simultaneous screening of many loci throughout the genome and the identification of loci that are putatively under selection [29]. As an example, genome scans were used to identify loci associated with morphological variation in European minnows (*Phoxinus phoxinus*) living in different habitat types [30].

In the present study, we focused on a fish species for which we hypothesized substantial morphological variation due to a variety of locally specific selective forces. The omnivorous cyprinid roach (*Rutilus rutilus*) occurs in many lakes and rivers of the European temperate zone [31]. Resource polymorphism in roach was recently demonstrated in two lakes [32]: individuals inhabiting the pelagic zone were streamlined and fed on zooplankton, whereas those living in the littoral zone had a deeper body and were feeding on benthic invertebrates [32]. The dominant feature of near-shore littoral habitats is their structural complexity, created by aquatic macrophytes or woody debris that accumulates in shallow water zones. Structural complexity enhances the biomass of prey organisms for omnivorous fish, as the physical structures provide a substrate for epiphytic algae, a primary food source for the invertebrate prey [33-36].

Much previous work has focused on structural complexity within lakes, but complexity also varies among lakes, with high complexity being a typical feature of shallow lakes in particular [37]. Shallow lakes with low phytoplankton abundance (“clear” lakes) can contain extended zones of structural complexity because the clear water allows light penetration and facilitates the growth of submerged macrophytes. In contrast, shallow lakes with an abundant phytoplankton community (“turbid” lakes) can have low structural complexity because the lack of light prevents macrophyte establishment. As a result, the differential selective forces operating in littoral and pelagic habitats within single lakes and inducing intralacustrine (i.e. within lake) divergence may also operate in an interlacustrine (i.e. among lake) comparison of populations from differently structured shallow lakes.

Here we examined morphology (geometric morphometrics), diet (gut content and stable isotopes) and genetic variation (AFLP) of roach in four shallow lakes in order to assess ecomorphological divergence and local adaptation among lakes. Two lakes were clear and had extensive macrophyte cover while two were turbid and had almost no macrophyte growth. We predicted that roach in clear lakes would feed mainly on benthic macroinvertebrates and have a correspondingly deeper body shape, and that roach from turbid lakes would be more planktivorous and exhibit a more slender shape. We also expected that morphological divergence would be further enhanced by exposure to different predators. The four lakes exhibited a gradient of predation pressure, measured as the proportion of piscivorous fish biomass in total fish assemblages, thus providing a basis to test for an association of body morphology with relative predation pressure. Finally, we tested whether the roach populations differed genetically between the lakes, and whether genetic markers were associated with the interlacustrine morphological divergence.

## Results

### Morphometric analysis

**Figure 1:**
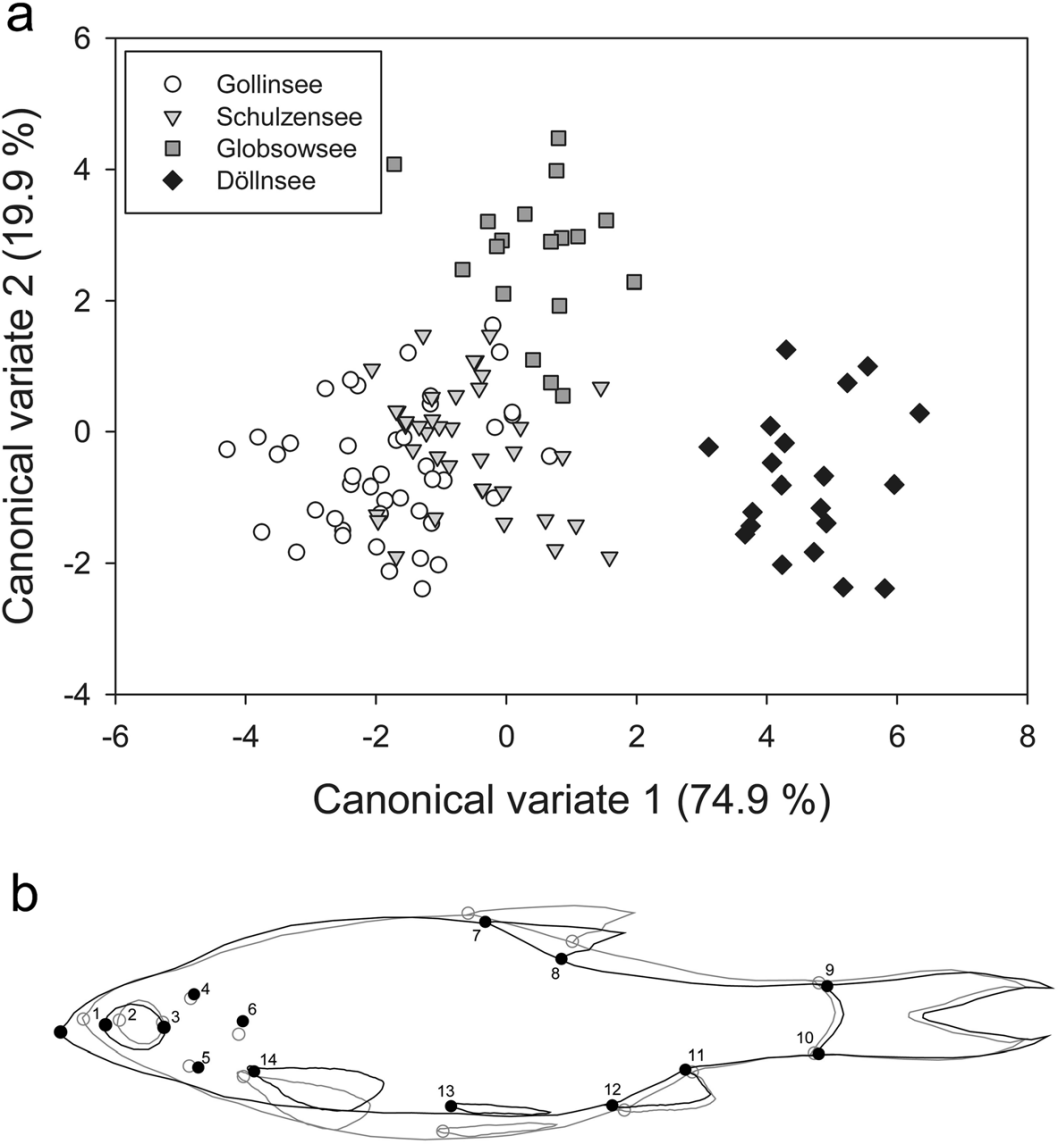
**Shape differences among populations of the four study lakes a) Results of CVA using geometric morphometric data to test for body shape differences among populations.** Bivariate plot show variation along the first two major axes of shape variation (canonical variates 1 and 2,% of predicted variance in brackets). **b**) Warped outline drawing (derived from thin-plate spline method, amplified by 250% to facilitate visualization) depict shape differences of fish from Döllnsee (black line), compared to the average shape of all fish (grey line). Positions of the 14 digitized landmarks used in geometric morphometric shape analysis are shown. 1: anterior tip of snout; 2: anterior margin of eye; 3: posterior margin of the eye; 4: dorsal margin of opercular (principal opercular bone); 5: ventral margin of opercular (principal opercular bone); 6: posterior margin of operculum; 7: posterior insertion of dorsal fin; 8: anterior insertion of dorsal fin; 9: superior insertion of caudal fin; 10: inferior insertion of caudal fin; 11: anterior insertion of anal fin; 12: posterior insertion of anal fin; 13: insertion of pelvic fin; 14: anterior insertion of pectoral fin.

Morphometric data were obtained from 119 individual roach: 43 from Kleiner Gollinsee (a turbid lake, hereafter referred to as Gollinsee), 38 from Schulzensee (clear), 18 from Globsowsee (turbid), and 20 from Kleiner Döllnsee (clear, hereafter referred to as Döllnsee). Fish from the four lakes differed significantly in their morphology, as revealed by permutation tests of Mahalanobis distances (Table 1). As visualized by graphical output of MorphoJ, landmarks 1 (anterior tip of snout), 7 (posterior insertion of dorsal fin) and 13 (insertion of pelvic fin) were most divergent among the four populations. As revealed from Canonical Variate Analyses (CVA), shape variations of canonical variate 1 (CV1, 74.9% of variance explained) were associated with the position of landmark 1 and body depth. CV2 (19.9% of variance explained) was associated with the location of the dorsal (landmarks 7 and 8) and pelvic fins (landmark 13) (Figure 1a). Roach from Döllnsee (clear) formed a distinct cluster in morphospace, located along the positive values of CV1. As visualized by warped outline drawings, roach from Döllnsee had a more elongated and fusiform body shape (Figure 1b). Furthermore, the dorsal fin was in a more posterior position relative to the average shape of roach. Variation in morphospace on CV2 was less pronounced and was driven by roach from Globsowsee (turbid, located along the positive values of CV2), characterized by a contraction of the dorsal fin and a shift of the pelvic fin toward the anterior part of the body.

**Table 1:** Pairwise distances between the group means in shape space

**Factor**	**Group comparison**	**Mahalanobis distance *D***	***P***-**value**
Lake	Gollinsee-Schulzensee	1.7470	0.0339
	Gollinsee-Globsowsee	4.1565	<0.0001
	Gollinsee-Döllnsee	6.8997	<0.0001
	Schulzensee- Globsowsee	4.1778	<0.0001
	Schulzensee- Döllnsee	6.5990	<0.0001
	Globsowsee-Döllnsee	9.4319	<0.0001

Results of DFA on size-corrected data are shown.

### Genetic (AFLP) analysis

AFLP genotypes were obtained for 183 roach (69 from Gollinsee, 70 from Schulzensee, 23 from Globsowsee and 21 from Döllnsee). We scored 447 loci, of which 331 (74%) were polymorphic over the four populations (Table 2). The percentage of polymorphic loci varied from 58.5 to 85.9% within the four populations (Table 2). Gene diversity (h_s_), as calculated by HICKORY, varied only slightly among lakes, ranging from 0.263 to 0.279 (Table 2). Bayesian statistics revealed a small but significant genetic differentiation among all four lakes (θ (HICKORY) = 0.03; 0.0246-0.0417 95% CI). Pairwise differentiation among the lake populations ranged from 0.0092 – 0.0702 with highest differentiation between populations of Globsowsee and Döllnsee and lowest between Schulzensee and Döllnsee.

**Table 2:** Genetic diversity of roach from four lakes of different structural complexity

**Lake**	***N***	**% polymorphic loci**	**h**_**s**_	**95% credibility interval of h**_**s**_	
				**Lower**	**Upper**
Gollinsee	69	62.0	0.275	0.268	0.280
Schulzensee	70	58.8	0.263	0.257	0.269
Globsowsee	23	85.9	0.279	0.270	0.286
Döllnsee	21	85.9	0.266	0.258	0.273
	Σ = 183	Total = 77.6	H_s_ = 0.271		

*N* = number of individuals analyzed, % polymorphic loci refers to the total of 447 loci analyzed, h_s_ = gene diversity with credibility intervals calculated by a Bayesian approach as implemented in HICKORY, H_s_ = the mean within-population expected heterozygosity.

Outlier analysis using BAYESCAN classified three loci (loci 31, 48 and 155; 0.91% of polymorphic loci examined) as outliers under divergent selection (probability *P* > 0.97 that the locus-specific component of F_ST_, α, is different from zero, corresponding to Posterior Odds > 32, estimated false discovery rate = 0.0084). Probabilities for the remaining 328 loci ranged from 0.064 to 0.572. The three outlier loci were present in all four populations, but with differing frequencies. Locus 48 dominated in Gollinsee (97%) and Globsowsee (88%), but was rarer in Schulzensee (28%) and Döllnsee (15%). In contrast, locus 155 dominated in Döllnsee (95%) and Schulzensee (78%), but was rare in Globsowsee (19%) and Gollinsee (11%). Locus 31 was highly frequent in Döllnsee (100%), Gollinsee (97%) and Schulzensee (91%), but less frequent in Globsowsee (31%). Linkage disequilibrium (LD; ARLEQUIN) was estimated for all possible pairs of the three outlier loci, but among the 12 (4 lakes × 3 pairs) pair-wise tests, only one locus pair in Gollinsee showed a significant linkage (*P* = 0.009, all other *P* > 0.23).

### Association of shape with predation pressure and genetic markers

**Figure 2:**
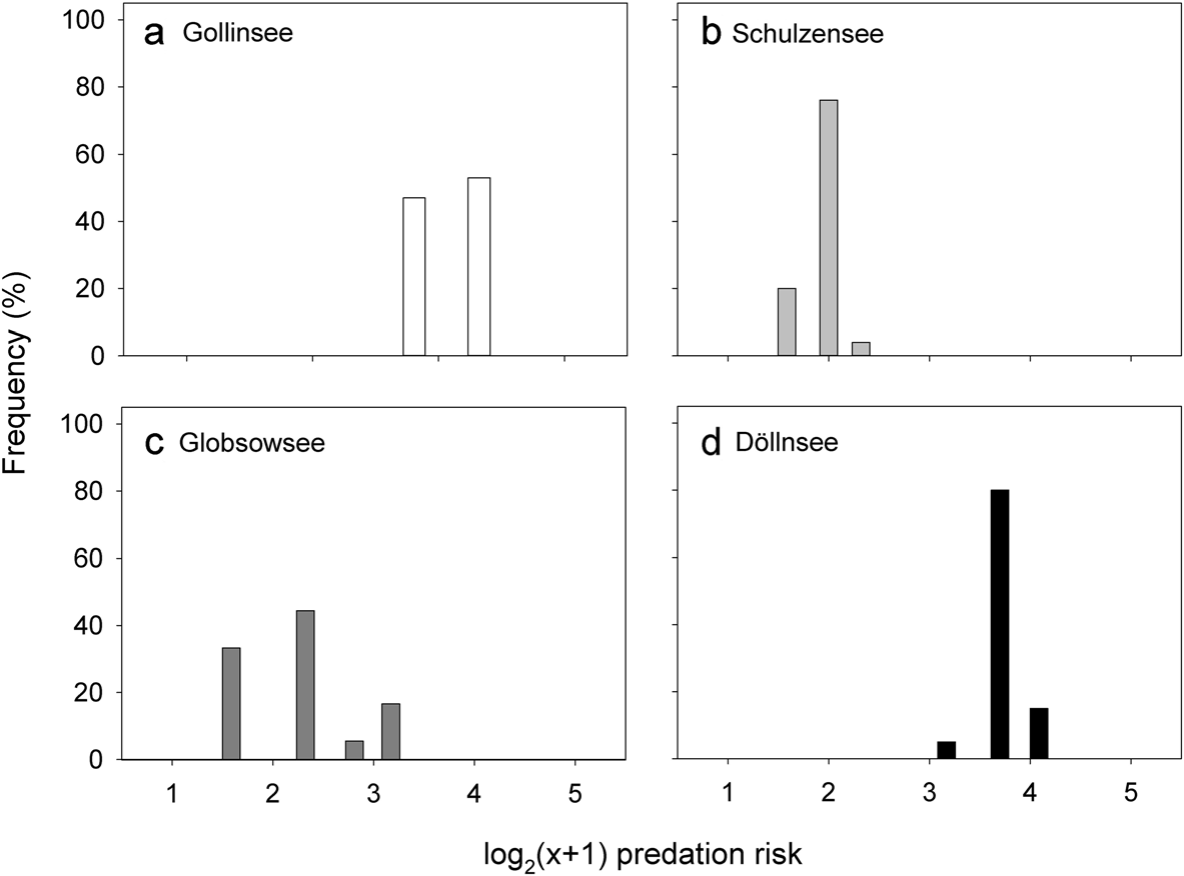
**Predation risk in roach of the four lakes studied.** Frequency distribution of individual predation risk (from 1 = low to 5 = very high) of roach in the four lakes studied (**a**-**d**). Predation score is a log_2_ (x + 1)-transformed composite of individual fish size and lake-wide piscivory.

**Figure 3:**
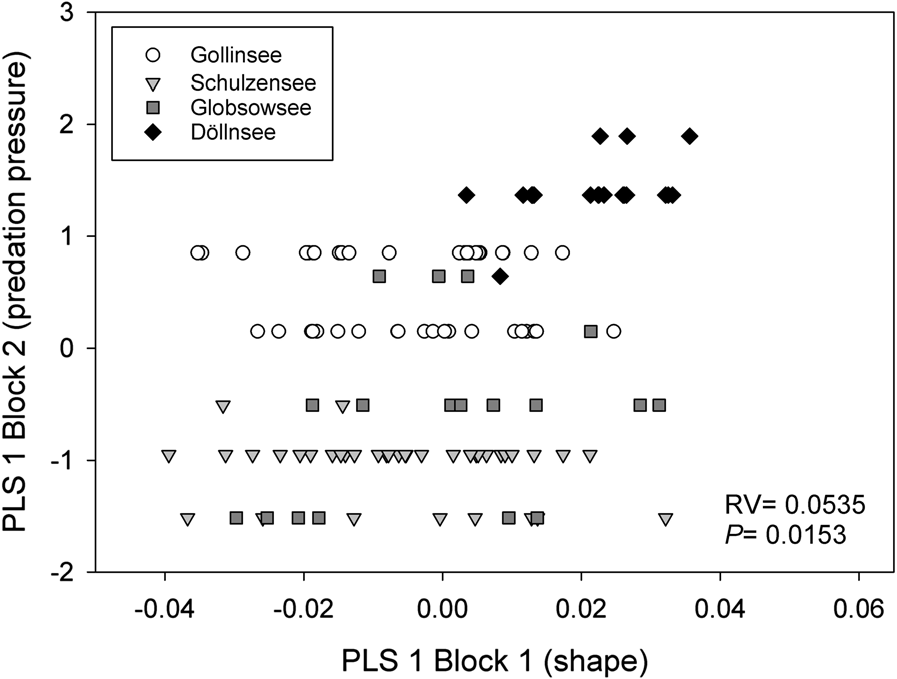
**Correlation of shape and predation pressure of four lakes of different structural complexity.** Results from PLS using size-corrected Mahalanobis distances as first block and predation pressure as second block are shown. *P*-value is obtained from permutation test against the null hypothesis of independence with 1000 randomization rounds.

Predation risk was highest for roach in Döllnsee (Figure 2d), because predator density was highest and roach were small in this lake. Lowest risk was in Schulzensee (Figure 2a), with intermediate risks in Gollinsee and Globsowsee (Figure 2b,c). Across the four lakes, shape and estimated predation pressure were significantly associated (two-block partial least square analyses (PLS), RV = 0.0533; permutation test against the null hypothesis of independence with 1000 randomization runs: *P* = 0.0153) (Figure 3). In lakes with higher predation pressure, the deviation in shape was attributable to body form and fin insertion.

Principal coordinate analysis (PCoA) of the 331 polymorphic AFLP loci aggregated 58.8% of variation along PCoA axis 1 (26.2%), PCoA2 (17.7%) and PCoA3 (14.9%). CV1 of shape analysis was significantly smaller in Globsowsee, Schulzensee and Gollinsee than in Döllnsee (GLM, F_7,85_ = 75.0, adjusted R^2^ = 0.85, *P* < 0.0001, coefficients of lake contrasts: *t* < −7.1, *P* < 0.0001), but aggregated genotypes (PCoA1) did not contribute to shape variations (coefficient for PCoA1, *t* = 0.36, *P* = 0.72). Interactions between PCoA1 and the lakes were likewise not significant (coefficients of interactions, *t* > 0.22, *P* > 0.83). Similarly, PCoA2 of genotypes and its interactions with lakes were not significant predictors of shape (CV1) (coefficient of PCoA2, *t* = −0.36, *P* = 0.34), but CV1 again significantly differed between Döllnsee and the other three lakes in this analysis (GLM, F_7,85_ = 78.5, adjusted R^2^ = 0.86, *P* < 0.0001; coefficients of lake contrasts, *t* < −7.1, *P* < 0.0001). General linear models on CV1 as the dependent dominant shape variable and lake and each of the three outlier loci as predictors confirmed that the genotype had no effect on shape of fish, because the effect of the outlier loci was not significant in any case (GLMs, F_7,85_ > 75.0, adj. R^2^ > 0.85, *P* < 0.0001; coefficients of the single outlier loci: locus 31, *t* = −0.27, *P* = 0.79; locus 48, *t* = 0.58, *P* = 0.56; locus 155, *t* = 0.43, *P* = 0.67).

### Roach diet and association with morphological data

**Figure 4:**
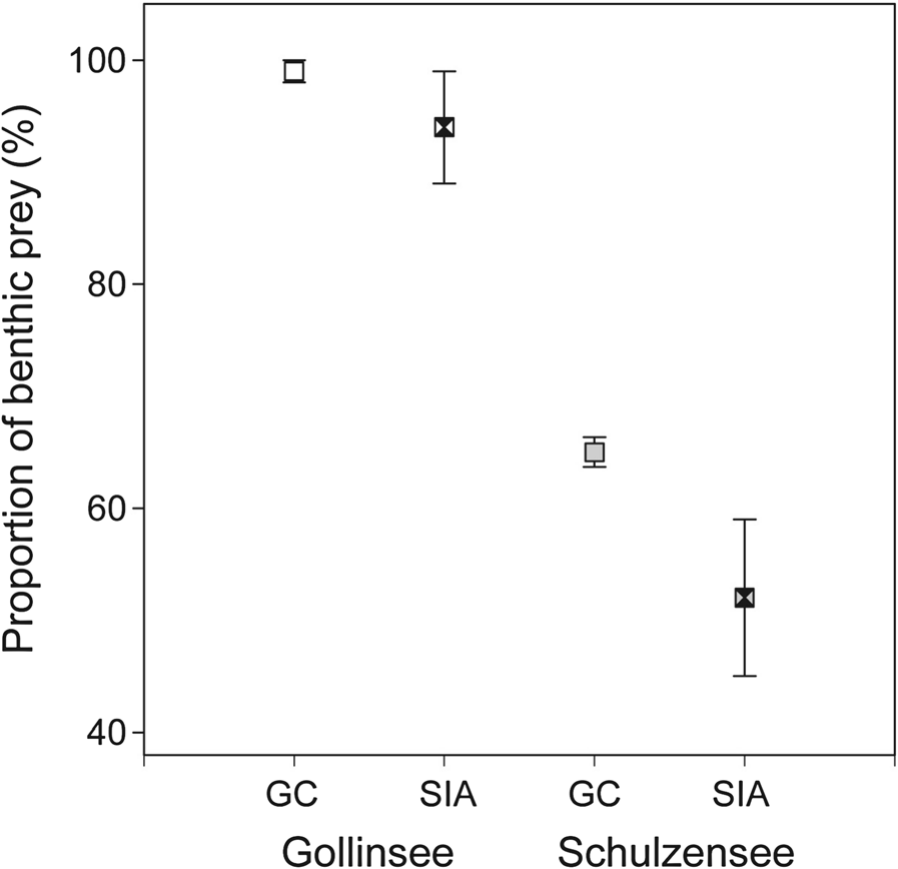
**Contribution of benthic prey to diet of roach.** Comparison of benthic contribution in diets of roach, revealed by gut content analysis (open symbols; depicted are mean values ± 95% confidence interval) and stable isotope analysis (shaded symbols; depicted are mean values ± 95% bayesian credibility interval) for roach from Gollinsee and Schulzensee.

Diet composition (gut content, GC, and stable isotope analysis, SIA) was analyzed from Gollinsee (turbid) and Schulzensee (clear). Roach from Gollinsee fed almost exclusively on benthic prey (Figure 4), as indicated by GC (mean 99% of prey) and the isotopic mixing model (mean 94%). In Schulzensee, the contribution of benthic and pelagic food sources was similar (Figure 4), although GC indicated a slightly higher use of prey from benthic habitats (mean 65%) than did the isotopic mixing model (mean 52%). The proportions of ingested benthic and pelagic prey items (as shown from GC) differed significantly between both lakes (Mann Whitney *U* = −5.033; *P* < 0.001).

PLS analysis of shape as the first block and gut contents of roach from Gollinsee and Schulzensee as the second block revealed no correlation of shape with diet composition (RV = 0.0475; permutation test with 1000 randomization runs: *P* = 0.66). Similarly, PLS with shape as the first block and isotopic ratios as second block did not show a significant correlation (RV = 0.0205; permutation test with 1000 randomization runs: *P* = 0.70).

## Discussion

Body morphologies differed in all four studied roach populations in shallow lakes, in particular with respect to overall body form and anterior-posterior location of the fins. Our analyses suggest that these morphological differences reflect a gradient in exposure to predation risk among the lakes, as shape and predation pressure were significantly correlated. Contrary to our predictions, fish morphology was not correlated with resource use. Therefore, we suggest that the strong morphological divergence among lakes in roach was primarily driven by differences in predation risk. The four populations exhibited small genetic differences. However, we did not find evidence that genetic differentiation and morphological variation among lakes were associated; suggesting that shape variation in roach did not have a heritable component detectable in our analysis.

Roach body shapes were most distinct in Döllnsee, the lake characterized by highest predator abundance. There are several examples for induced morphological defenses in fish, including a plastic response to achieve a size refuge against predators limited by gape-size [19-21]. Here, we observed a more slender body and a more caudal position of the dorsal fin. In contrast to morphological changes that prevent handling of prey by predators [19-21] we consider this body form to be advantageous for flight. To escape a predator attack, most fish show a similar fast-start behavior of sudden, high-energy swimming bursts, called a “C-start” [38-40]. During a C-start, fish bend into a “C” shape (stage 1) and then produce a propulsive stroke of the caudal region in the opposite direction (stage 2). This is facilitated by a slender body form and a relatively shallow anterior body and head region, as these contribute minimally to drag [41-43]. Thrust can be increased if the dorsal fin is in a caudal position [41]; however, Domenici et al. [44] demonstrated higher speed and acceleration in Crucian carp of deeper body forms, attributable to a high percentage of muscle mass. Therefore, there seems to be a trade-off between a streamlined body that reduces drag and a deeper body of high muscle mass that increases burst.

A previous study compared morphology of one-year-old roach raised under experimental conditions in the presence and absence of pike (*Esox*) predators [45]. The main differences were changes of dorsal fin displacement and morphological responses similar to our findings. The authors suggested that these morphological changes affect swimming speed and manoeuvrability in roach [45]. Contrary to our study, they detected a wider anal fin in roach exposed to predators. Adaptations to faster swimming seem advantageous, as roach were found to increase activity and even show the tactic of jumping out of the water when threatened by a predator [46]. Comparable adaptations to faster predator escape as a response to high predator abundances were also found in Western mosquitofish (*Gambusia affinis*) [43] and in *Galaxias platei*[47], where caudal regions were enlarged to enhance burst performance. A recent review [48] concluded that most of the observed predator-induced morphological defenses are purely a by-product of swimming activity. Prey swim less when predators are present, resulting in lower feeding rates or decreased metabolism, leading to reduced growth. We cannot exclude that variation in roach morphology between the lakes reflects differences in swimming activity (in response to differing predation risk); however, roach from Döllnsee, where predation risk was highest, had morphologies that appear better adapted to rapid and extended swimming, which contrasts the conclusion that predation risk always induces reduced swimming activity of prey.

Body morphology was not associated with structural complexity in the lakes created by macrophyte coverage, with little evidence for an association of morphology and diet composition in this omnivorous species. Gut content and stable isotopes both suggested that benthic invertebrates dominated the diet of roach in the two lakes analyzed, whereas shape data significantly differed between these populations. Similar studies of fishes mainly focused on intralacustrine (within-lake) morphological divergence and resource polymorphism, with specialization on benthic food usually associated with a preference for littoral habitats with high structural complexity. Intralacustrine resource polymorphism driven by intraspecific competition has been studied in roach of two Swedish lakes [32]. Benthivorous individuals from the littoral zone had a deeper body compared to planktivorous individuals from the pelagic zone. Similarly, pumpkinseed sunfish produce molluscivorous and a planktivorous morphotypes to decrease intraspecific competition [49,50]. Ours is the first study of interlacustrine (among-lake) divergence of body morphology in roach of which we are aware, but similar morphological divergence between lakes has been reported for populations of three-spined sticklebacks in Alaska, USA [51]. Very shallow lakes, where most of the stickleback habitat is structurally complex, were inhabited by individuals of the benthic form, characterized by a deeper body that favors maneuverability, whereas the limnetic form with a narrower body that reduces drag during prolonged swimming was found in deeper lakes with large areas of open waters [41].

A possible explanation for the lack of correlation between resource use and body shape here could be the spatial dimension of the systems studied. The four lakes we studied were small (< 0.2 km^2^) and relatively shallow. Roach may be able to easily exploit the resources from all habitats because near shore and offshore sites are usually less than 100 m apart. Furthermore, seasonal and annual changes in resource abundance require flexibility in resource use, perhaps counteracting any selective advantage gained by specialization. A high diet flexibility of roach is supported by the fact that guts of roach in the lake with almost no macrophytes (Gollinsee) contained even more benthic prey than those from fish in the lake with intermediate macrophyte coverage (Schulzensee). A more balanced diet composition with resources from both littoral and pelagic origin was obtained by applying the mixing model based on stable isotope analysis. Therefore, in systems where resources are spatially dispersed and temporally variable, selection may favor morphological shapes that support omnivory rather than discrete morphotypes specialized on a particular prey and habitat type.

Many previous studies found morphological changes to be phenotypically plastic [19,52], although a few studies have demonstrated a heritable component [43,53,54]. In our dataset, we did not find any statistical association of shape differences with aggregated or outlier-specific genotypes among the lakes. However, the three outlier loci occurred in all populations, although with differing frequencies, suggesting that the allele frequencies of these loci are not the result of neutral genetic drift. There is some inherent uncertainty in AFLP studies, such as false scoring of alleles due to differences in PCR efficiency (false positives) or peak overlap (false negatives) [55]. Furthermore, reduced precision during sample preparation due to sampling mix-up, pipetting or contamination may cause genotyping errors [56]. Nevertheless, we have no indication that false positives had a strong influence on our results, because the reproducibility was 98% within the randomly chosen repeated 17.4% of samples.

The proportion of outlier loci (0.91%) fits within the range of reported outliers of former studies (0.4 – 24.5%) [28]. In a comparison of three available statistical programs, BAYESCAN (the method we used here) showed the lowest rates of false positives [57], and hence we consider the three loci found in our study to be reliable (see also the very low false discovery rate estimated by BAYESCAN for our data). AFLP outlier loci have been linked to morphological diversification in other studies of fish, including European minnows in lake and stream habitats [30] or in sympatric lake whitefish (*Coregonus clupeaformis*) ecotypes [58]. A similarly strong association between morphotype and genotype could not be confirmed for roach in our study. It is possible that our AFLP markers simply failed to include genomic regions that are associated with the shape variations between the lakes. Alternatively, the lack of association between genotype and phenotype may suggest a plastic response to predation pressure. Compared to fixed genetic controls, plastic responses to environmental pressures are advantageous when predatory regimes are variable [59]. Strong interannual variability of predation pressure is likely in the communities analyzed here because these small and shallow lakes are characterized by frequent fish kills during strong winters that can substantially reduce predator abundance [60].

Overall, the extent of genetic divergence between populations is determined through a mixture of adaptive and neutral processes [61]. Local adaptation is achieved through environmental filtering of genotypes [62], whereas neutral processes include drift-migration equilibrium wherein landscape isolation plays a role in reducing gene flow (e.g., isolation by distance, IBD) [63]. We did not find evidence for a dominance of neutral processes and IBD in our data. The geographical distance between the four lakes is very limited (<35 km), and the two lakes situated only 4 km apart (Gollinsee and Döllnsee) exhibited intermediate genetic divergence (θ = 0.0266). Thus, we conclude that interlacustrine morphological diversification of the four roach populations is mainly led through processes related to local environments of each lake (in particular predator abundance) rather than neutral process due to geographic isolation between the lakes.

## Conclusions

We observed divergent morphologies in omnivorous fish populations among four shallow lakes. Our results support previous studies suggesting that predation risk may play a more important role than habitat and diet in shaping body morphology of this prey species. In the case of roach inhabiting small and shallow lakes, we consider these morphological changes advantageous for fast escapes and thus directly cued by predation. Furthermore, we did not find evidence that the observed morphological divergence had a heritable component.

## Methods

### Study areas and sampling

The four lakes are situated in northeast Germany; about 100 km north of Berlin (see Table 3 for more details). All lakes are small and shallow, and characterized as either clear, structurally complex lakes with some coverage by submerged macrophytes (Schulzensee and Döllnsee) or turbid, phytoplankton-dominated lakes without submerged macrophytes (Gollinsee and Globsowsee). Geographical distances between the lakes range from 4–35 km. They are not connected by waterways and the small size of their inlets and outlets allows dispersal only in years with extremely high water levels.

**Table 3:** Characteristics of the four lakes included in the study

**Lake**	**Area (km**^**2**^**)**	**Mean depth (m)**	**No. fish species**	**Coverage of submerged macrophytes (%)**	**Relative abundance of piscivorous fish (%)**
Gollinsee	0.033	1.7	7	0	3
Schulzensee	0.039	2.2	5	22	0.3
Globsowsee	0.148	2.8	7	< 1	12
Döllnsee	0.250	4.1	12	51	23

Fish were caught using Nordic multi-mesh gill nets (length 30 m, height 1.5 m; 12 mesh sizes from 5–55 mm; Lundgrens Fiskredskapsfrabrik AB) or electrofishing with 200–400 V DC current (4–8 electrofishing aggregates EFG 4000, EFGI 4000; Bretschneider Spezialelektronik, Breitenbrunn, Germany) with anodic handnets (4–4.5 m long, 40 cm diameter, 6 mm mesh size) during summer and early autumn 2010. After capture, fish were measured (mm total length, TL) and weighed (g wet mass, wm) and subsequently stored on ice. In total, 185 roach (69 from Gollinsee, 70 from Schulzensee, 25 from Globsowsee and 21 from Döllnsee) were processed. Due to methodological problems with some individuals, the number of fish per analysis was slightly lower.

### Morphometric analysis

Body morphology of individual roach was examined using a landmark-based geometric morphometric method [64]. Fish were photographed from the left side in a standardized way using a Nikon DX40 and a 28 mm lens, which represents a normal lens when considering the sensor size of the camera. Optical distortions were minimized by using a normal lens, a moderate distance to the specimen, and an aperture close to the critical aperture. Individuals were placed in a bowl of fine white gravel to obtain a straight position and the fins were stretched out to make the fin base visible. A few specimens with injuries on the left side were photographed from the right side and then the photographs were digitally flipped horizontally prior to analysis. After taking the photo, individuals were cut open to determine the sex. The shape data of the two sexes did not differ significantly (Mahalanobis *D* = 1.0761; *P* = 0.53), therefore sexes were pooled for further analyses. All roach were at least two summers old, an age where roach in lakes at this latitude are sexually mature [65].

Digital photographs were transferred to TPSdig2 (all TPS- programs http://life.bio.sunysb.edu/morph) and 14 landmarks were digitized. To diminish measurement error, digitizing was always performed by the same person. Haphazardly chosen individuals were repeated and results were compared to determine reliability of the results. We checked for outliers with the “Find outliers” function of the software MorphoJ. TPSSmall was used to determine whether the amount of variation in shape in our data set was small enough to permit statistical analyses in the linear tangent space approximated to the non-linear Kendall's shape space. For all further analyses, MorphoJ was used. Landmark configurations were aligned by Procrustes Superimposition [66] to minimize effects of translation, rotation and scaling. Shape data were size-corrected using a regression of shape (i.e. Procrustes coordinates) on size (i.e. log centroid size) for each lake separately. Residuals obtained from this regression were used for all further analyses. A Discriminant Function analysis (DFA) and a Canonical Variate analysis (CVA) were used to assess significance of shape differences between groups. A pairwise comparison between all four lakes was conducted (Globsowsee vs. Döllnses vs. Gollinsee vs. Schulzensee).

### Genetic (AFLP) analysis

AFLP analysis followed Vos et al. [67] with some exceptions noted below. Total genomic DNA was extracted from dorsal fin tissue using DNeasy Tissue Kits (Qiagen, Hilden, Germany) following the manufacturer’s protocol. DNA quality and concentration were inspected using a spectrophotometer (Nano Drop 1000, Thermo scientific, Wilmington, USA). DNA (250 ng per sample) was digested for 6 h at 37°C using 2 U MseI and 10 U EcoRI (all enzymes from New England Biolabs, Ipswich, USA), 2 μg BSA, 2 μL EcoRI buffer, and 16 μL sterilized dH_2_0. Ligation was then performed by adding 2 U T4 DNA-Ligase, 1 μL MseI adapter, 1 μL EcoRI adapter, 4 μL T4 DNA ligase buffer (10×), 4 μL ATP and 7.5 μL sterilized dH_2_0 at 16°C overnight. Preselective amplification was carried out using one additional base on each primer (MseI + C and EcoRI + A, oligonucleotides from Metabion International AG, Martiensried, Germany) and a thermocycler (VWR DuoCycler, VWR International GmbH, Darmstadt, Germany) programmed with a denaturation at 92°C for 20 seconds, an annealing at 56°C for 30 seconds, and an elongation at 72°C for two minutes for a total of 20 cycles, followed by a final extension for 30 minutes at 60°C. Selective amplification was conducted under the same thermal protocol using four primer pairs where three additional bases were added at 3’-end of each primer. On EcoRI-primers, four different fluorescent dye labels were attached (6FAM™-labeled primers from Metabion; VIC®-, NED™- and PET®-labeled ones from Applied Biosystems, Life Technologies, Darmstadt, Germany). Initially, 20 different primer-pair combinations were tested, and four gave best results (Table 4). Fragments of selective amplification were denatured at 95°C for 5 min and then cooled on ice for 10 min, before they were separated on an ABI 3500xl capillary sequencer (Applied Biosystems) with an internal size standard (GeneScan-600 LIZ®; Applied Biosystems). Correct fit of the size standard was visually inspected for all electropherograms.

**Table 4:** Primer combinations of selective amplification

**Primer combination**	**MseI**-**Primer**	**EcoRI**-**Primer**	**Number of loci**
1	MseI + CAG	EcoRI + ACT + 6FAM™	133
2	MseI + CAG	EcoRI + ACA + VIC®	139
3	MseI + CAG	EcoRI + AAG + NED™	107
4	MseI + CTA	EcoRI + AAG + PET®	68

All primers had 3 additional base pairs at the 3’end. EcoRI-Primers were also labeled by a fluorescent dye at the 5’end.

Signal processing and binning was conducted using Genemapper™ V.4.1 (Applied Biosystems). Presence (1) or absence (0) of fragments were scored between 50 and 450 bp using a peak intensity threshold > 100 relative florescent units. A bin width of 1 bp and a max peak of 1.5 bp yielded optimization and fragments were scored between 50 to 450 bp. A complete repetition of a random 17.4% of the samples yielded 97.8% reproducibility.

The input data files for statistical analyses were prepared by AFLPDAT [68]. AFLP-SURV [69] was used to calculate the frequency of polymorphic fragments. To obtain information on genetic structure within and among all four populations, genetic differentiation was calculated by using a Bayesian approach (θ) as implemented in HICKORY [70], using 20,000 burn-in iterations and 100,000 final iterations. AFLP loci potentially under selection (outlier loci) were identified using the BAYESCAN software [71] by assuming that loci influenced by directional selection show larger genetic differentiation than neutral loci, and loci that have been subjected to balancing selection show a lower genetic differentiation. This software uses a Bayesian likelihood method by reversible jump Monte Carlo Markov Chains. From F_ST_ calculations, posterior probabilities of two models are estimated: one model including selection on a certain locus (the locus-specific component of F_ST_, α, is different from zero), the other one not. The Posterior Odds (ratio between these probabilities) provides a detection level for a locus to be under selection. We retained loci with *P* > 0.97 (Posterior Odds > 32) as outliers presumably under directional selection. These calculations were run only with the 331 polymorphic AFLP loci. We further used ARLEQUIN 3.5 to test for pairwise linkage disequilibrium (LD) of the outlier loci, using 1000 steps in the Markov chain and a dememorization of 1000 steps.

### Predation pressure

To approximate the predation pressure by piscivores on roach for each lake, lake-specific abundances of piscivorous fish were multiplied with the individual, size-dependent predation risk of roach from that lake. For Globsowsee, Schulzensee and Gollinsee, relative abundances of piscivorous fish were obtained from standardized gill net catches (6–8 benthic multimesh gill nets set over night in autumn). We included perch (>15 cm TL) (see [72] for a definition of piscivory in perch) and pike as piscivorous fish species. In the fourth lake (Döllnsee), destructive gill-net fishing could not be conducted because the lake is equipped with a 3D-telemetry system for *in-situ* studies of fish behavior. Therefore, we used information on fish assemblage composition from gill-net samplings conducted in previous years [73,74]. The proportion of piscivores in Döllnsee calculated from earlier samplings is conservative because the lake has been heavily stocked with pike during the last years, and hence roach presumably experience an even greater predation pressure. The lake-specific relative abundances of piscivorous fish were ranked from highest (predator abundance = 4) to lowest (predator abundance = 1) (Figure 2). Predation risk of prey fish is size-dependent due to gape limitation of piscivores [21]. Therefore, predation risk of individual roach was ranked according to total length, with TL ≥ 25 cm: predation risk = 0; TL ≥ 20-25 cm: predation risk = 1; TL ≥ 15-20 cm: predation risk = 2; TL ≥ 10-15 cm: predation risk = 3; TL < 10 cm: predation risk = 4 (Figure 2). The individual predation risk was ultimately multiplied with the rank among lakes with respect to relative abundances of piscivores. Therefore, the smallest roach in Döllnsee had the highest relative risk (4 × 4 = 16), whereas all roach > 25 cm TL had a risk of zero, independent of lake of origin. For all subsequent analyses, the obtained product for predation risk ranging from 0 to 16 was log_2_(x + 1)-transformed to achieve a near-linear distribution.

### Gut content and stable isotope analysis

Detailed data on roach diet composition was obtained from roach in Gollinsee (*N* = 42) and Schulzensee (*N* = 42). In the laboratory, roach guts were removed and stored in 5% formaldehyde for subsequent GC. Individuals with empty stomachs were removed and the anterior third of the gut was examined under a stereo microscope and the volume proportion (equivalent to area proportion at uniform width) of each prey category observed in the sample was estimated to the nearest 10%, adapted from Windell [75]. The observed prey items were grouped into two categories, namely benthic prey (trichoptera, gastropods, isopods, chironomid larvae, bryozoans, algae and detritus) and pelagic prey (cladocerans, copepods, water mites, ostracods, rotifers, and chaoborid larvae). Eleven fish with empty guts were removed prior to analysis. A non-parametric Mann–Whitney *U* test was conducted to compare the percentage of benthic and pelagic prey between the two lakes.

For SIA of carbon (^13^C/ ^12^C) and nitrogen (^15^ N/ ^14^ N) from roach (*N* = 46 from Schulzensee and *N* = 47 from Gollinsee, including fish with empty guts), a small sample of dorsal muscle tissue was excised from each fish. To obtain baseline values of potential diet groups for the omnivorous roach, macroinvertebrate samples for SIA were collected in April and June from various sites in the littoral zone using a sweep net. Zooplankton samples for SIA were taken by hauling zooplankton nets (55 μm and 100 μm) vertically through the entire water column several times during summer. Samples were taken to the laboratory and transferred to clean tap water to allow the animals to void their guts overnight. Macroinvertebrates were then sorted into taxon groups and zooplankton samples were filtered (100 μm filters). All samples were dried at 60°C for 48 hours, then ground to a fine powder using a mortar and pestle and material of about 0.5 mg dry mass was loaded into tin cups. Carbon (%C) and nitrogen (%N) content and stable isotope ratios of C (δ^13^C) and N (δ^15^N) were analyzed on a FlashEA 1112 elemental analyzer coupled to a Thermo Finnigan DELTA ^Plus^ Advantage mass spectrometer (Thermo Electron Corporation, Waltham, MA, USA) at the University of Jyväskylä, Finland. Stable isotope data are expressed in the conventional delta notation as the relative difference between ratios of samples and international standards (PeeDee Belemnite for δ^13^C, atmospheric N for δ^15^N). Analytical precision (mean SD from in-house standard) for each run was always better than 0.3‰ for δ^13^C and δ^15^N.

To calculate relative contribution of benthic and pelagic food sources to the diet of roach in both lakes, the Bayesian isotope mixing model package SIAR (Stable Isotope analysis in R) [76] was used in R version 2.12.0 [77]. Fractionation factors were set to 0.4 ± 1.3‰ for δ^13^C and 3.4 ± 1.0‰ for δ^15^N, as suggested in a previous study [78]. The mean isotopic value of the zooplankton samples of each lake was taken as an end point for pelagic δ^13^C and δ^15^N values. Littoral δ^13^C and δ^15^N end points were calculated from the mean value of the macroinvertebrates, containing trichoptera, isopoda, chironomidae and gastropoda. These taxa represent the most abundant littoral macroinvertebrates consumed by roach.

### Association of shape data with genetic data and ecological variables

To reduce the dimensionality of the genotype information from 331 polymorphic AFLP loci, we performed Principal Coordinate analysis (PCoA) by applying the standardized covariance method. Genetic distance between two individuals was calculated as the sum of loci with different character states, a method that automatically discards monomorphic loci. These analyses were conducted in GenAlEx 6.5 [79,80]. To examine association of shape with genotype, general linear models (GLM) between the dominant canonical variate (CV1) of shape data as dependent variable and lake as group variable were calculated. We used contrasts between Döllnsee (the lake with the most deviating shape) and the other three lakes. The first two axes of PCoA on AFLP-data and their potential interactions with lake origin were then used as continuous predictors. Using these GLMs, we tested whether the shape of roach differed among the four lakes, and whether the aggregated lake-specific genotype contributed to this differentiation. In a second step, we used only the three outlier loci and their interactions with lake origin as binary predictors of shape. With these GLMs, we tested whether the genotype outliers were directly associated with the differences in shape between the lakes. GLM analyses were conducted in R version 2.15.1. [77].

A two-block partial least square analysis (PLS), as implemented in MorphoJ, was used to study the association of shape and ecological variables at the level of individual roach. In all calculations, only those individuals for which all the respective variables were available to facilitate paired comparisons were included. All variables used in the second blocks were adjusted to standard deviate $b=\left(x_{\mathrm{ij}}-\bar{x}\right)/s_{i}$; where s_i_ is the standard deviation of row or column i. To study correlation with predation pressure, shape was used as a first block and relative predation pressure as second block. To correlate shape with diet composition, PLS were calculated using percentage of ingested diet items obtained from GC or isotope ratios. A permutation test against the null hypothesis of independence (1000 randomization runs) was used for all PLS, as implemented in the software MorphoJ.

## Competing interests

The authors declare that they have no competing interests.

## Authors’ contributions

KS carried out the morphometric, molecular genetic and stable isotope analyses, conducted most of the statistical analyses and drafted the manuscript. KW established some of the molecular genetics protocol and helped carrying out the genetic study. JS helped carrying out the stable isotope study and helped with the isotopic mixing model. TW carried out the gut content analysis. MTM provided laboratory facilities and discussion on evolutionary processes. TM together with KS conceived of the study, participated in its design and coordination, carried out some statistical analysis and helped to draft the manuscript. All authors read and approved the final manuscript.
